# Supplementary material for: Characterizing limit order books in call auctions of a stock market
Source: PLoS One. 2025 Jul 7;20(7):e0327430. doi: 10.1371/journal.pone.0327430 (PMC12233305; doi:10.1371/journal.pone.0327430)
Supplement: S1 Fig — (PDF) [file pone.0327430.s001.pdf]

Supporting Information
Characterizing limit order books in call auctions of a stock market

Shota Nagumo[1*], Takashi Shimada[1,2†],

1 Department of Systems Innovation, Graduate School of Engineering, The University of Tokyo, Tokyo, Japan
2 Mathematics and Informatics Center, The University of Tokyo, Tokyo, Japan

* shota.nagumo.0614@gmail.com
† shimada@sys.t.u-tokyo.ac.jp

S1 Fig: Fitting orders by the piecewise linear functions

We have fitted the cumulative sell orders and buy orders $N^A(x)$ and $N^B(x)$ with the hyperbolic tangent functions, but we use a different function as the fitting function in order to validate whether we can obtain the consistent result as the hyperbolic tangent functions. As an example, we use the piecewise linear functions $\widehat{N}^A$ and $\widehat{N}^B$ as the fitting functions of $N^A(x)$ and $N^B(x)$ as the following equations.

$$\widehat{N}^A(x) = \max\left[\gamma^A(x-\theta^A)+\eta^A,\ \eta^A\right], \quad \widehat{N}^B(x) = \max\left[\gamma^B(\theta^B-x)+\eta^B,\ \eta^B\right].$$

Here, $\gamma^A, \theta^A, \eta^A, \gamma^B, \theta^B$, and η^B are the fitting parameters. Although the number of fitting parameters in the piecewise linear functions is larger than the hyperbolic tangent functions, the fitting error of the former is larger than the latter. Therefore, the hyperbolic tangent functions are relatively good fitting functions of cumulative orders compared to the piecewise linear functions.

When we define the medians of sell order and buy orders α and β which satisfy $\widehat{N}^A(\alpha) = N^A/2$ and $\widehat{N}^B(\beta) = N^B/2$ and define the average width $\bar{\omega}' = (\omega^{A'} + \omega^{B'})/2$ where $\omega^{A'} = \alpha - \theta^A$ and $\omega^{B'} = \beta - \theta^B$ are satisfied, we can observe the relation between the median spread $\alpha - \beta$ and the width $\bar{\omega}'$ as shown in Supporting Fig 1. We can see that there is a large cluster of stocks which are distributed around the straight line and that below the cluster, there is another cluster which is formed mainly by the stocks of the companies whose net profits are larger than 10 billion yen. This is consistent with the results obtained using the hyperbolic tangent functions.

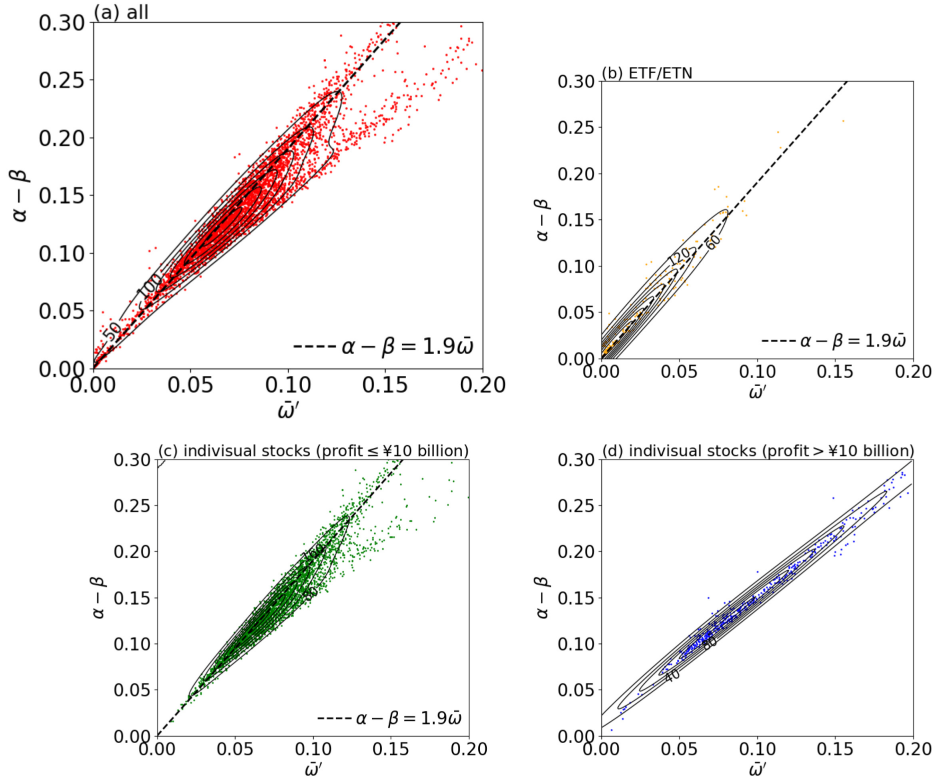

Supporting Fig 1. Scatter plots when using the piecewise linear functions as the fitting functions of (a) All the stocks, (b) ETF/ETN, (c) Individual stocks whose net profits are less than 10 billion yen, and (d) Individual stocks whose net profits are more than 10 billion yen, on the plain of width ($\bar{\omega}$) and spread ($\alpha - \beta$). The density contours are plotted by the solid lines. The dashed line is $(\alpha - \beta)/\bar{\omega}' = 1.9$.
