# Supplementary material for: Characterizing limit order books in call auctions of a stock market
Source: PLoS One. 2025 Jul 7;20(7):e0327430. doi: 10.1371/journal.pone.0327430 (PMC12233305; doi:10.1371/journal.pone.0327430)
Supplement: S2 Fig — (PDF) [file pone.0327430.s002.pdf]

Supporting Information
Characterizing limit order books in call auctions of a stock market

Shota Nagumo[1*], Takashi Shimada[1,2†],

1 Department of Systems Innovation, Graduate School of Engineering, The University of Tokyo, Tokyo, Japan
2 Mathematics and Informatics Center, The University of Tokyo, Tokyo, Japan

* shota.nagumo.0614@gmail.com
† shimada@sys.t.u-tokyo.ac.jp

S2 Fig: Using the 2019 data

When we use the 2019 data, we can obtain the result that is consistent with the 2022 data. That is, as shown in Supporting Fig 1, there is a large cluster of stocks which are distributed around the line: $\alpha - \beta = 3\bar{\omega}$, and below the cluster, there is another cluster which is distributed around a sub-linear curve. The latter cluster is formed mainly by the stocks of the companies whose net profits are larger than 10 billion yen.

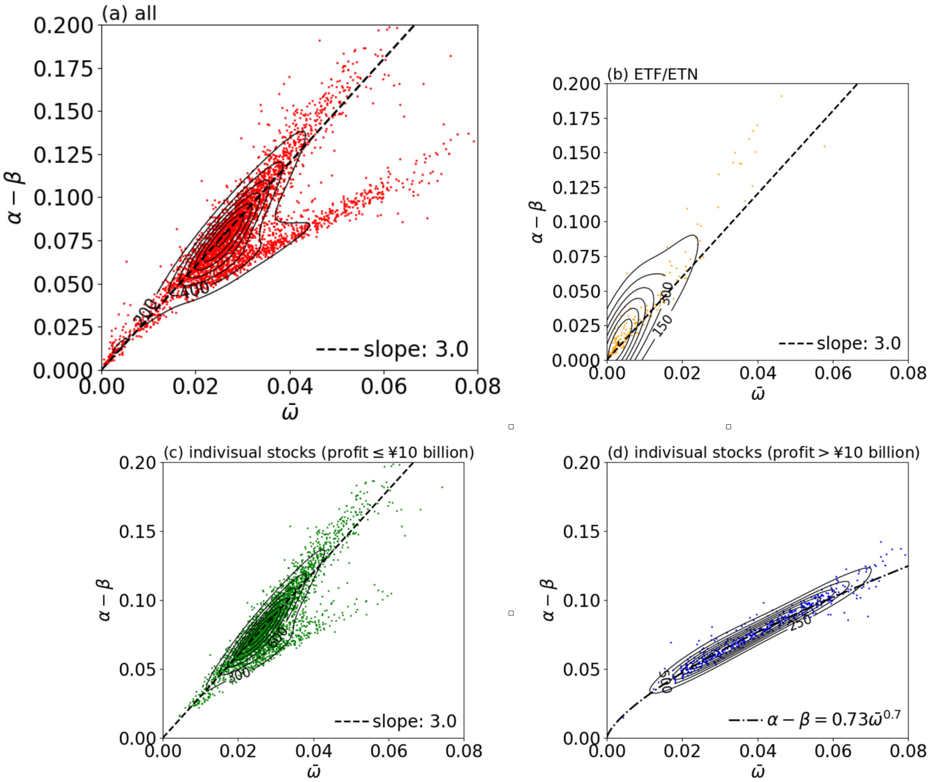

Supporting Fig 1. Scatter plots when using the 2019 data of (a) All the stocks, (b) ETF/ETN, (c) Individual stocks whose net profits are less than 10 billion yen, and (d) Individual stocks whose net profits are more than 10 billion yen, on the plain of width ($\bar{\omega}$) and spread ($\alpha-\beta$). The density contours are plotted by the solid lines. The dashed line $(\alpha-\beta)/\bar{\omega}=3.0$ corresponds to the equal execution ratio ($\widehat{v}=4\%$) line, and the dash-dotted curve $\alpha-\beta=0.73\bar{\omega}^{0.7}$ is for the eye guide.
