# Supplementary material for: Characterizing limit order books in call auctions of a stock market
Source: PLoS One. 2025 Jul 7;20(7):e0327430. doi: 10.1371/journal.pone.0327430 (PMC12233305; doi:10.1371/journal.pone.0327430)
Supplement: S3 Fig — (PDF) [file pone.0327430.s003.pdf]

Supporting Information
Characterizing limit order books in call auctions of a stock market

Shota Nagumo[1*], Takashi Shimada[1,2†],

1 Department of Systems Innovation, Graduate School of Engineering, The University of Tokyo, Tokyo, Japan
2 Mathematics and Informatics Center, The University of Tokyo, Tokyo, Japan

* shota.nagumo.0614@gmail.com
† shimada@sys.t.u-tokyo.ac.jp

S3 Fig: The BBO spread vs the width

Clusters 1, 2, and 3 have been discovered on the plain of width $\bar{\omega}$ and median spread $\alpha - \beta$. However, if we substitute the median spread by conventional BBO spread $a_0 - b_0$, such clustering structure disappears as shown in Supporting Fig 1. This illustrates the importance of taking the shape of the limit order book apart from best ask (bid) to better capture the state of the stock market.

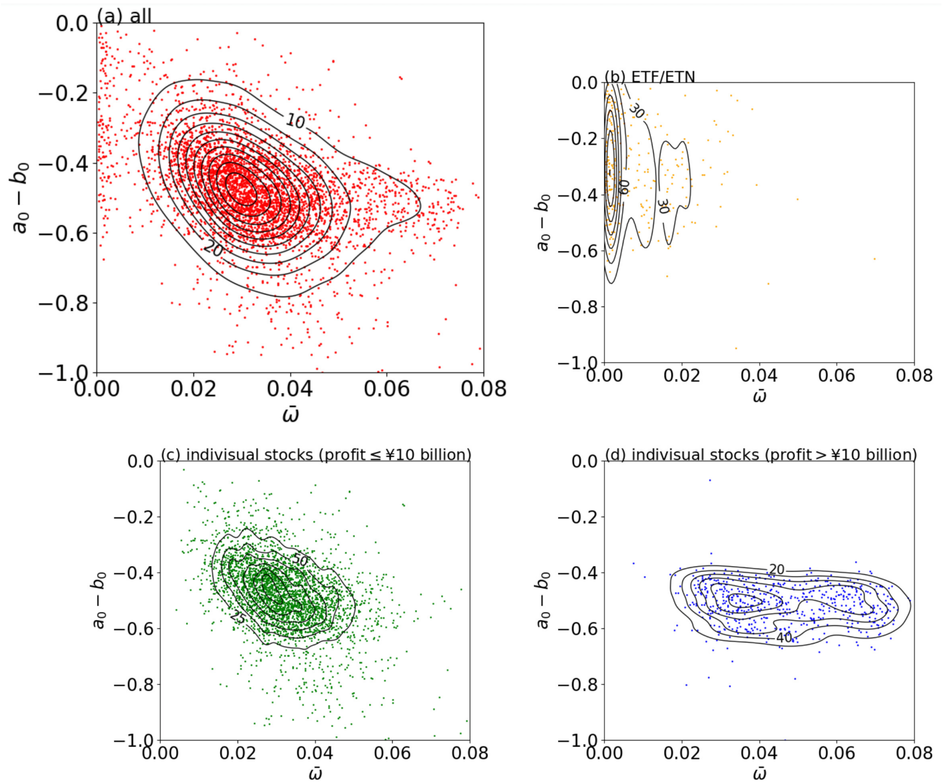

Supporting Fig 1. Scatter plots of (a) All the stocks, (b) ETF/ETN, (c) Individual stocks whose net profits are less than 10 billion yen, and (d) Individual stocks whose net profits are more than 10 billion yen, on the plain of width ($\bar{\omega}$) and BBO spread ($a_0 - b_0$). The density contours are plotted by the solid lines.
